# An Address on the Fiftieth Anniversary of the Graduation of Drs. Clawson and Smith1Delivered before the Medical Society of the County of Schuyler, at the Gle Springs, Watkins, N. Y., March 30, 1911.

**Published:** 1911-05

**Authors:** Charles G. Stockton

**Affiliations:** Buffalo, N. Y.


### An Address on the Fiftieth Anniversary of the Graduation of Drs. Clawson and Smith[1]

By CHARLES G. STOCKTON, M.D.
Buffalo, N. Y.

IT is on an occasion like the present one when the professional friends of two cherished physicians and the County Society of which they are members unite to congratulate them on the attainment of the fiftieth anniversary of their graduation into medicine that recollections arise of a kind that enable us to see with greater clearness the relations that the physician bears to society and to human advancement.

But to begin with, I wish to extend my appreciative thanks to the Medical Society of the County of Schuyler for the courtesy that brings me here today, and for the opportunity provided me of bearing to your honored guests the congratulations and good wishes of the Faculty of the Medical Department of the University of Buffalo. The richest possessions of the college are the traditions, the high ideals that distinguished alumni have carried away with them. When the graduates show by years of successful work that they have developed the ideals received in Buffalo and thus evince their appreciation of and their loyalty to the school, it becomes a realisation of the relationship that should exist between the college and the alumni—of which the college is justly proud.

Personally, I wish to congratulate Dr. Smith and Dr. Clawson upon their good fortune in having enjoyed half a century of professional activity; and furthermore, upon the fact that they have lived during the most wonderful decades, so far as medicine

---

1. Delivered before the Medical Society of the County of Schuyler, at the Gle Springs, Watkins, N. Y., March 30, 1911.

is concerned, that the world has ever seen, or probably ever will see. I find that the young men who are now entering the profession have but the dimmest conception of the changes that have taken place in medicine since these gentlemen, whose anniversary we celebrate, began the practice of their profession. When they entered their work, the teachings of Owenbrugger and Laennec were comparatively recent, and there were many physicians at that time who failed to understand the art of percussion and auscultation in physical examinations. In those days there were men whose practice was based upon the teachings of Benjamin Rush in medicine and Sir Ashley Cooper in surgery. There were men who still failed to realise the truth of the great Louis observations in the differentiation of fevers. They were beginning to appreciate that there was a difference between typhus and typhoid, but they were not quite clear where the line should be drawn. There were mighty discussions carried on as to antiphlogistic treatment. Antimony was still held to be an indispensable boon, blood-letting still had the field, and as to calomel, half the profession agreed with Rush that it was "the Samson of materia medica," and the other half replied, "yes, this is so because it has slain its thousands and tens of thousands." Anesthesia had not as yet lost its novelty, and therefore the domain of surgery was comparatively narrow. It is hard to realise that the children of Dr. Morton, the discoverer of anesthesia, are as yet relatively young people, and it is difficult to believe that the great levers, by means of which disease is studied and modified today, have for the most part come into our possession within the life-time of men now living.

These observations have been so often made that they have come to be merely conventional, or "bromide" remarks. But Mr. Roosevelt has reminded us that the perfectly obvious things are those which it is most useful to keep in mind and most profitable to reflect upon. It ill becomes us to acclaim too proudly the greatness of our times, when the gods must yet look upon our failures with pity if not with amusement. But, if we find difficulty in 1911 in successfully grappling with the problems set before us, how much more difficulty must have faced our predecessors? If we read faithfully the pages of medical history, I think that the conclusion will be reached that there have been great physicians throughout all time, as we know it. There has been an enormous amount of medical lore that has disappeared from human annals. That which has been written is a poor record of what has been practised. Probably the great principles have been handed down without having undergone any very striking change, but I think that we fail to recognise how much men could accomplish by the intelligent application of certain of these principles even when deprived of the knowledge of others. It is not so much the number of tools of which an artisan has knowledge as it is his familiarity and his skill in the use of them. Just as we look with wonder at a miniature temple which some genius has carved out with a jackknife, so we may well be astonished at the good that has been accomplished in comparatively remote times by simple means when guided by good judgment and great intelligence.

If in the past physicians were embarrassed because of a want of resources with which to become familiar, on the other hand, we of today, are embarrassed by the multiplicity of resources with which we never can hope to become more than moderately acquainted with. The best medicine today is of necessity carried on by a group of men, for the reason that no one man has time or ability to learn to play all the instruments of the orchestra, and I think it is a matter that might well be debated whether the physician of today, unassisted by his fellows, would be more successful in dealing with the average medical problem than was the doctor of a century ago, provided he was a man of ripe understanding. I hope not to be misunderstood in saying this, for it is in no pessimistic spirit that it is uttered. Merely let us consider the disadvantage of attempting to deal with innumerable, half digested resources. Professor Miner was accustomed to say that if he were allowed half a dozen drugs and his instrument case he would feel competent to practise all the medicine and surgery that was good for anything. Think of a man saying that today! when the really important items in our armamentarium are practically beyond the ability of one's memory to recall.

The immortal Pasteur who, about fifty years ago, was struggling to establish the truth by his cure for the silkworm disease, and thus save millions to France annually, while he was beset with abuse, misunderstanding, depreciation and all sorts of attacks, solaced himself by recalling that it took three hundred years to fully establish in the minds of Europeans that potatoes were fit for human food. Late in the 18th century potatoes were held to be the cause of leprosy. Their popularity waxed and waned, and in France the blessing which, as a cheap food, they brought to the multitude, was not established until their social position, if you please, was established through the fact that Louis XVI, in order to kill the prejudice, wore the flower of the potato in his buttonhole. It seems to require centuries for the full conversion of mankind to any doctrine, and then, if I may be pardoned the Hibernianism, he fails to stay converted. For illustration, consider the deplorable case of vaccination. In spite of logic, of fact, of observation and statistics, there are thousands of misguided and almost fanatical people, even in enlightened America, who are fighting against compulsory vaccination. But one must not take the attitude of Schopenhauer; on the whole, the outlook is promising.

Whenever I feel discouraged over the failure of high-minded professional effort to win the confidence of wrong thinking people, I recall an experience in the little French seaport of Boulogne-sur-Mer, not many years ago. I saw an attractive monument in a little square in front of the town hall, and soon discovered that it was erected in honor of Jenner. On closer examination I found upon it an inscription which may be translated as follows: "This memorial was erected by the Geographical Society of Paris and by the people of Boulogne-sur-Mer in honor of Edward Jenner, the discoverer of vaccination, who sent his assistant with vaccine to this town to overcome smallpox which was then raging, although at that time there existed war between France and England." It seems to me that this is about the best epitome of the spirit of our profession which has come to my acquaintance. While the powers of France and England were bent on destruction, not counting the blood, the wounds, the privation, the pillage and broken homes,—the simple-minded English doctor, in the spirit of humanity, did what he could to stay the plague of smallpox in the army of his country's enemy; and that which was almost equally remarkable and beautiful was the spirit in which this act was received and acknowledged by the gallant French people. So long as there dwells in humanity nobility like this, it is well worth while going on in the weary struggle in attempting to get a pure water supply, to exterminate contagious diseases, and to provide the community with pure food.

I congratulate myself on having been for a third of a century a practitioner of medicine and of having had the opportunity of seeing the extraordinary revolution which has followed the discoveries of Louis Pasteur. It has been given to no other man to do for the race that which Pasteur accomplished as the result of marvelous intelligence and intuition, of enormous effort and with beautiful modesty. I love to recall his charming personality, his simple, cordial manner, so lacking in false pride and so full of interest for every truth.

The gentlemen whose anniversary we celebrate today have lived to see the suggestion, the adoption and the fulfillment of the germ theory of fermentation and of disease. Surely that has been a great reward.

But it should not be forgotten, and therefore it is well to rehearse the violent and depressing opposition against which this doctrine had to struggle. The men of middle age remember the scorn with which their seniors spoke of the "germ theory" of disease. For years it was the butt for the sarcasm not only of pathologists but of the surgeons as well. A man less high-minded and devoted than Pasteur would have been crushed by the ridicule and contumely heaped upon him, and this notwithstanding the fact that with remarkable rapidity he accomplished a triumphant success in one problem after another, several of them having immediate practical results in preserving and making profitable the industries of his countrymen. Von Liebig, the greatest of German chemists, declined to the last to give credence to the new explanation of fermentation. This caused pain to Pasteur, and as late as 1870, he went to the trouble of visiting Von Liebig at Vienna, with the hope of demonstrating the truth of his doctrine. Von Liebig received Pasteur courteously, but emphatically declined even to discuss the problem with him.

It is interesting to recall the long, long struggle regarding the nature of tuberculosis. It seems but yesterday that the journals were debating the validity of the experiments of Villemin, the discoveries of Koch, the practical work of Klebs. It is interesting to compare the state of mind of those not very distant days with the sentiment of today which prompts hundreds of thousands of teachers and workers to spread the lesson of the contagiousness of tuberculosis. What a marvelous spectacle it has been, this campaign of invasion of the mysteries of tuberculosis, that immortal enemy of the human race. It has been dramatic for the conflict is tragic, and in spite of all our work, we realise that the campaign has only fairly begun. The profession does not yet realise its responsibility on the question of dealing with incipients, and the community, case-hardened from long familiarity, is slow in taking steps for the isolation of advanced cases.

One after another, the secrets of infection are being unraveled, and to us has been vouchsafed the great joy of recognising the numerous discoveries which have been crowded into comparatively few years, and yet each one of these is epoch making. The real glory of the accomplishments would have been better appreciated had these resounding discoveries been scattered along the centuries. The announcements of great finds have been so crowded together that we have become somewhat blasé in experiencing the unfolding of great truths and are unequal to the fitting display of our appreciation and our gratitude for that which science has accomplished in the last half century.

It is just about fifty years ago that Pasteur accomplished his first great work in explaining the nature of fermentation, and

I wish I might live to see the name of this most helpful of mankind suitably honored throughout the civilised world. For although he did not survive to grapple with each individual problem, Pasteur foresaw and predicted the explanation and the cure of those infections which have been so praiseworthily dealt with by his successors. Tuberculosis, cholera, leprosy, bubonic plague, diphtheria, erysipelas, malaria, yellow fever, puerperal fever, typhus fever, malta fever, typhoid and relapsing fevers and syphilis, not to extend the list too far, are revealed to us in their true nature and are no longer hidden foes, are no longer spoken of as the visitation of Providence, nor attributed to Demonology. Perhaps we shall survive to greet the recognition of the cause of cancer. When that discovery is announced the older men will feel that they have lived to witness the fulfillment of their professional aspirations, and the younger ones will feel the stimulation that will lead them into even more enthusiastic work. We are living in times of tremendous deeds and of immortal men.

To realise that this is true is to bring solace to the minds of thousands of humble, conscientious practitioners, the illy requited and poorly appreciated teachers of ignorant and whimsical humanity, whose obstinancy and obstructive characteristics lead us, when we are fatigued and old and half sick, to feel despondent. However, when one reviews the last five decades and compares its achievements with those of all preceding history, I think that one may feel that it has been worth while to devote these years to the study and practice of medicine and to feel the assurance that the physicians of the future will reward the ghostly procession of the sons of Esculapius which reaches back to those shadowy days when Apollo gazed upon the sparkling waves around his island home of Delos.

